# Acute Flaccid Myelitis Outbreak

**DOI:** 10.15844/pedneurbriefs-29-12-7

**Published:** 2015-12

**Authors:** J. Gordon Millichap

**Affiliations:** 1 Division of Neurology, Ann & Robert H. Lurie Children's Hospital of Chicago, Chicago, IL; 2 Departments of Pediatrics and Neurology, Northwestern University Feinberg School of Medicine, Chicago, IL

**Keywords:** Flaccid Myelitis, Spinal Gray Matter, Motor Cranial Neuropathy, Enteroviruses

## Abstract

Investigators from the University School of Medicine, Salt Lake City, Utah, report an outbreak of acute flaccid myelitis (AFM) occurring in 2014-2015 in several States and reported to the CDC.

Investigators from the University School of Medicine, Salt Lake City, Utah, report an outbreak of acute flaccid myelitis (AFM) occurring in 2014-2015 in several States and reported to the CDC. The disease was localized to the gray matter of the spinal cord. Eleven children, ages 13 months to 14 years (median, 9 years) presented in the intermountain West with extremity weakness (n=10) or cranial neuropathy (n=1), varying in severity and without apparent etiology. Maximum paralysis occurred within 4 days of onset. Seven children had a prodrome consisting of headache, nausea, vomiting, or cough one to 7 days before onset of weakness; 5 had intermittent, low-grade fever. None described sensory loss. Only one required intubation for respiratory failure. MRI showed T2 hyperintensities involving the anterior horn gray matter of the spinal cord; four children also had T2 hyperintensities involving the brainstem or cerebellar nuclei. CSF pleocytosis in 7 children ranged from 7 to 170 leukocytes/uL. PCR enteroviral and herpesvirus tests were negative. Respiratory film array was positive for influenza AH3 in one child and parainfluenza 2 in another; rhinovirus was detected in a nasopharyngeal swab in one child, thought to be coincidental. Treatments included IV immunoglobulin, corticosteroids, and plasma exchange, all having no beneficial effect, and 9 of 10 (90%) having residual motor deficits at follow-up. As of July 2015, 120 cases of AFM have been reported to the CDC, and no cause has been identified. [1]

COMMENTARY. AFM is a unique type of flaccid paralysis in children, distinct from Guillain-Barre syndrome (GBS) and transverse myelitis (TM). GBS is an ascending paralysis, associated with sensory symptoms, characteristic CSF findings, and favorable prognosis. TM has a prominent sensory loss whereas AFM has focal, poliomyelitis-like spinal cord paralysis with minimal or no sensory symptoms [1]. AFM should be considered in the differential diagnosis of children who present with GBS or TM-like symptoms, and especially in those with persistent weakness.

Testing for enteroviruses is extensive because of their known association with AFM. Of 11 patients presenting with AFM and/or cranial nerve dysfunction during an enterovirus D68 outbreak in Colorado, 9 had brain stem lesions, most commonly involving the pontine tegmentum, and 10 had longitudinally extensive lesions in the central gray matter of the spinal cord. MR imaging showed enhanced ventral cauda equina nerve roots in 4 patients, and ventral cervical nerve roots enhanced in 3. Neuroimaging findings were similar to those described in outbreaks of viral myelitis caused by enterovirus 71 and poliomyelitis [2, 3].
